# Unlocking Probiotic Potential: Physicochemical Approaches to Evaluate Probiotic Bacterial Adhesion Potential to the Intestinal Tract

**DOI:** 10.1002/mnfr.202400705

**Published:** 2025-01-23

**Authors:** Thị‐Thanh‐Trúc Phùng, Sébastien Dupont, Laurent Beney, Julie Chanut, Thomas Karbowiak

**Affiliations:** ^1^ L'institut Agro, Université Bourgogne Europe, INRAe, UMR PAM Dijon F‐21000 France

**Keywords:** bacterial adhesion, comprehensive evaluation, gastrointestinal tract, physicochemical approaches, probiotics, surface properties

## Abstract

Bacterial adhesion in the gut is critical to evaluate their effectiveness as probiotics. Understanding the bacterial adhesion within the complex gut environment is challenging. This study explores the adhesion mechanisms and the adhesion potential of five selected bacterial strains (*Escherichia coli*, *Lactiplantibacillus plantarum*, *Faecalibacterium duncaniae*, *Bifidobacterium longum*, and *Bifidobacterium longum* subsp. *infantis*) at the initial stages when bacterial cells arriving in the gut, using different physicochemical approaches. Bacterial morphology, rheology, and surface properties were evaluated. Surprisingly, previous methods such as bacterial adhesion to hydrocarbon and the interfacial tension between bacterial suspensions and mineral oil did not fully capture the bacterial adhesion to intestinal mucus. Consequently, this study introduced a novel approach to assess bacterial adhesion to mucus, based on contact angle measurements, calculation of surface tension, and work of adhesion. Interestingly, both small and large intestinal mucus are rather hydrophilic, and thus highly hydrophilic bacteria such as *E. coli* and *B. infantis* tend to adhere better. Additionally, a multicriteria evaluation of bacterial adhesion to the gut, from the bulk liquid transport stage until the irreversible adhesion, was proposed. *E. coli* and *B. infantis* demonstrated the highest overall adhesion potential in the intestinal tract, followed by *Lpb. plantarum*, *B. longum*, and *F. duncaniae*, respectively. This work contributed original physicochemical approaches to comprehensively examine bacterial adhesion in the gut.

## Graphical abstract

From the oral cavity to the establishment of bacterial adhesion in the intestinal tract, probiotic cells traverse 4 main stages: (1) bacterial transport, (2) reversible adhesion, (3) irreversible adhesion, and (4) biofilm formation. Each stage involves distinct mechanisms that govern the adhesion potential of bacteria to the intestinal mucus, which needs to be separately evaluated. This work proposes novel physicochemical approaches to assess the initial adhesion stages of bacteria in the gut. In this context, comprehensive methodologies for a holistic understanding of the adhesion mechanisms were introduced, facilitating the studies of probiotic functionality.

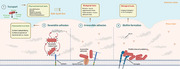

## 1 Introduction

Bacterial adhesion is the first step which potentially leads to proliferation and thus colonization of probiotics in the gut of the host [1]. It is, therefore, one of the key mechanisms to study when assessing the effectiveness of probiotics. However, the gastrointestinal lining is a multicomponent and highly complex system [2, 3]. This challenges the comprehensive evaluation of bacterial adhesion in such an environment.

**FIGURE 1:**
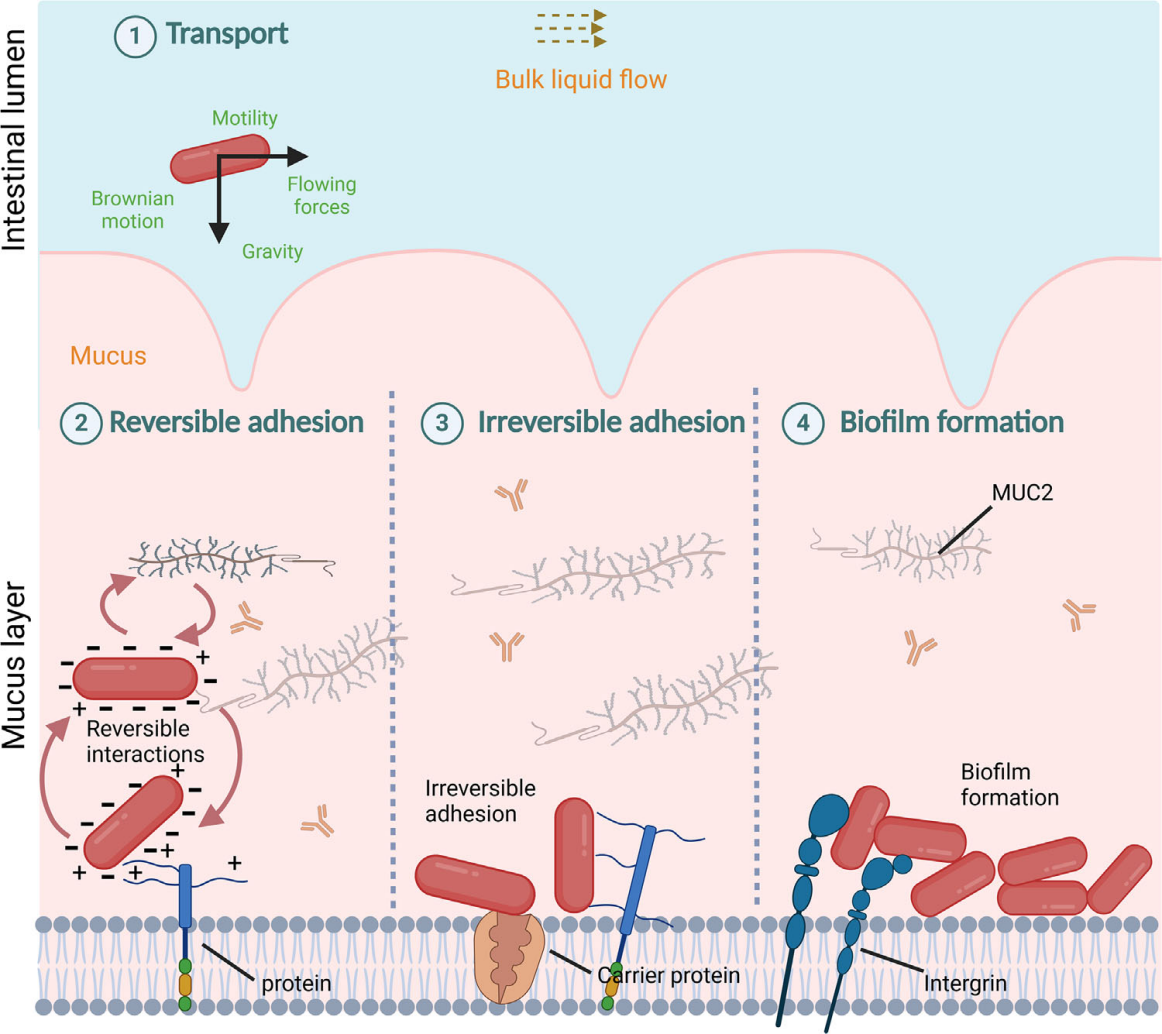
From the oral cavity until bacterial adhesion is established in the intestinal tract of the host, probiotic cells have to pass through different stages including (1) bacterial transport, (2) reversible adhesion, (3) irreversible adhesion, and (4) biofilm formation. At the first stage of bacterial transport, bacteria are exposed to the bulk liquid flow, where their mobility is affected by their cell motility, morphological properties, and rheological properties. Upon approaching closer to the substratum, bacteria interact with the mucus layer, where irreversible adhesion can happen between bacterial cells themselves and/or with mucus components. These interactions are affected by the Lewis acid–base interactions, Lifshitz–van der Waals, and electrostatic forces according to the extended DLVO theory proposed by van Oss [6]. Once bacteria reach the intestinal substratum, irreversible adhesion can be formed between bacterial cells and the surface substrates such as proteins, integrins, etc., and thereby, biofilm can be produced.

The bacterial adhesion in the intestinal tract can be divided into four different stages: bacterial transport; reversible adhesion; irreversible adhesion; and in some cases, biofilm formation [4, 5], as described in Figure 1.

Initially, probiotic cells are transported freely in the intestinal cavity, where they are exposed to the bulk liquid flow. Probiotic cells encounter various environmental factors that force them to perform active or passive movements in the planktonic medium or bulk liquid upon approaching the intestinal surface [4]. At this stage, the mobility of bacterial cells is mainly influenced by the thrust of the bulk liquid flow, in addition to the gravity effect, the Brownian motion, and the motility of bacteria. In this context, the morphological attributes of bacterial cells are believed to play a key role in bringing them closer to the intestinal substratum, as larger cell dimensions increase the impact of the flowing forces. Also, the analysis of bacterial movement in the liquid phase can reveal the self‐motility of bacteria, which can facilitate the initial adhesion potential in the gut.

When a certain closeness is established between the bacterial cells and the substratum, the mucus outer layer, in this case [3, 7, 8], reversible adhesion can occur [5]. This stage of bacterial–surface interaction has previously been explained using the Derjaguin–Landau–Verwey–Overbeek (DLVO) theory, which describes the net interaction between colloidal particles and a flat surface [5, 9, 10]. However, the DLVO theory assumes both the substratum and the colloidal particle surfaces are chemically inert, which is not applicable to the complex, biologically active surfaces of bacterial cells and intestinal components. To address this limitation, van Oss proposed the extended DLVO (XDLVO) theory, incorporating polar interactions, including Lewis acid–base interactions, alongside the original Lifshitz–van der Waals (LW) and electrostatic (EL) forces [6]. The magnitude of polar interactions between bacterial cells and surfaces, as described by the extended DLVO theory, can significantly exceed that of the EL and LW interactions [11]. The strength of these polar forces, which involve Lewis acid–base interactions, is highly dependent on the hydrophobic or hydrophilic nature of both the bacterial cell surface and the substrate. In some cases, these polar interactions can be up to 10–100 times stronger than the combined EL and LW forces [11]. Accordingly, the measurement of the hydrophobicity of bacterial cells and mucus surface can reveal the adhesion potential of bacteria to the intestinal tract.

It is important to note that the intestinal epithelium is covered by a thick layer of intestinal mucus, that provides a protective function, keeping gut microflora as well as probiotics adhered mainly to its outer layer [3, 7, 8]. Once bacterial cells reach the mucus surface, irreversible adhesion can be established between bacteria and the specific mucus components. This specific adhesion of bacteria is facilitated by adhesins present on the outer layer of bacterial cell surface and receptors on the intestinal surface, including intestinal epithelial surface molecules (IESMs) and enzymes [12, 13, 14]. The adhesion of bacteria to IESMs has been performed using the cell adhesion assay, where bacteria were subjected to adhere on a microtiter plate‐coated layer of a single component such as epithelial cell lines like Caco‐2 or specific proteins like bovine serum albumin (BSA), Mucin type 2 (MUC2), etc. [15, 16]. More comprehensively, the specific adhesion of five probiotic strains has previously been tested on a mucus layer, where all the components existing in intestinal mucus were taken into account [17].

In some cases, a strong specific adhesion can result in a biofilm formation and thereby, the colonization in the gut. The adhesion of probiotic bacteria to the intestinal surface has previously been investigated using scanning electron microscopic (SEM) observation [17]. This imaging technique serves as an additional confirmation of the adhesion potential of bacteria in the intestinal tract.

This study aims to comprehensively elucidate the initial bacterial adhesion potential to the intestinal tract using quantitative physicochemical approaches. The main focus is based on the investigations of the impact of morphological properties, rheological properties, as well as the surface properties of bacterial cells to their overall adhesion potential to the gut mucosa. In addition, this study also critically reinvestigates the previously proposed hypotheses regarding the role of bacterial and intestinal mucus hydrophobicity in this process. This work also contributes novel physicochemical approaches to determine the adhesion potential of probiotic bacteria to the intestinal tract. Finally, a multicriteria evaluation of bacterial adhesion potential from the initial stage of passive transport until irreversible adhesion is proposed, by combining with the results obtained from our previous study about bacteria‐mucus irreversible adhesion using ex vivo approaches [17]. This facilitates a full‐scale assessment of the adhesion phenomenon of probiotic bacteria which previous adhesion methods have not been fulfilled.

## 2 Materials and Methods

### 2.1 Bacterial Culture

Five bacterial strains were used: *Escherichia coli* K‐12 TGI (Microbiology Laboratory, L'institut Agro, Dijon, France), *Lactiplantibacillus plantarum* 103151T (Pasteur Institute, Paris, France), *Bifidobacterium longum* BAA‐999TM (BB536) and *Bifidobacterium longum* subsp. *infantis* 15697TM (S12) (both from ATCC, VA, USA), and *Faecalibacterium duncaniae* A2‐165 DSM17677 (DSMZ, Braunschweig, Germany). It is important to note that *E. coli* K‐12 TGI is not classified as a probiotic. However, this strain was selected as they have a distinctive morphology, as well as surface properties, thus facilitate the evaluation of adhesion. Bacteria were cultured at 37°C according to a previously described protocol [17]. Cells were harvested at the late exponential phase and washed thrice with PBS by centrifugation (3000 × *g*, 6 min).

### 2.2 Sample Preparation for SEM Observation

Freshly prepared bacterial culture, with identical optical density measured at a wavelength of 600—OD_600_ = 1.0, was deposited on a silicium wafer for 5 min. The samples of *F. duncaniae* A2‐165, *B. longum* BB536, and *B. infantis* S12 were prepared under anaerobic conditions. All the samples were washed twice with PBS, and fixed with 4% paraformaldehyde and 2.5% glutaraldehyde for 24 h. Samples underwent dehydration through an ethanol gradient (30–90%, v/v), followed by critical point drying in liquid CO_2_ (EM CPD030, Leica microsystems, Austria). After gold sputter coating (5 nm) using an ion sputter coater (Q150T ES plus, Quorum, UK), samples were examined using FEG‐SEM (Hitachi SU8230) at 3 kV.

### 2.3 Sedimentation Test

Four milliliters of fresh bacterial culture diluted to OD_600_ = 0.6 was deposited into a glass cuvette (Hellma Macro Cuvette‐6030‐OG‐ Fisher Scientific—Dublin, Ireland) for sedimentation. The initial optical density (OD_o_) measured at time 0 (*t*_0_) of the bacterial culture was used as a reference. The experiments were performed at ambient conditions (25 ± 1°C). This temperature was selected in order to obtain comparable results with other physicochemical tests conducted owing to the fact that not all the experiments in this study could be done at 37°C, which is the temperature of the human gut. After about 4 h (*t*_1_) of sedimentation, the final optical density (OD_1_) of the bacterial culture was measured. The sedimentation rate over 1 h was calculated as follows:

(1) $$\mathrm{Sedimentation}\;\mathrm{rate}\;\left(\%\right)=\frac{1-\frac{OD_{1}}{OD_{0}}}{t_{1}-t_{0}}\times 100$$

### 2.4 Size Distribution of Bacteria

The size distribution of bacteria was carried out using a granulometry MasterSizer 3000 (Malvern Panalytical, Malvern, UK) at ambient conditions (25 ± 1°C). The measurement is based on the scattering of the laser light by liquid‐suspended particles. Before analysis, the instrument was calibrated following several washing steps and then the reservoir was loaded with PBS solution, the reference media used to disperse bacteria. About 5 mL of fresh bacterial suspension (OD_600_ = 1.0) was added into the reservoir under a continuous stirring speed of 1280 rpm. The size distribution of bacterial particles was reported in volume density (%), representing the volume fraction of bacterial particles in different sizes and number density (%), representing the degree of concentration of bacterial particles. Each experiment was done in triplicate and their mean value was calculated for interpretation of the data.

### 2.5 Dynamic Viscosity and Density of Bacterial Culture

The fresh bacterial culture was diluted to OD_600_ = 0.6 for all measurements. Dynamic viscosity tests were conducted using the rolling‐ball viscosimeter Lovis 2000 (Anton Paar GmbH, Graz, Austria). The density of bacterial suspension was measured by the u‐shape densitometer when the sample passed by before getting through the capillary. The measurements were conducted at a constant temperature of 25°C, at ambient conditions. The experiment was done in triplicate and their mean was calculated for interpretation of the data.

### 2.6 Physical Destabilization of Bacterial Suspension

The fresh bacterial cultures were diluted to OD_600_ = 0.6 for all measurements. The physical stability of the bacterial suspensions was studied at 25°C, and ambient conditions, using Turbiscan LAB system (Formulaction—Toulouse, France) based on multiple light scattering measurements. The instrument operated at a wavelength of 600 nm. The duration of the test was set for 4 h. A measurement was taken every 5 min. The acquired data were analyzed using the software TurbiSoft‐Lab‐2.2.0.82‐5. T% represents the transmittance of laser light, indicating the percentage of light that passes through the bacterial suspension. Meanwhile, ΔBS represents the difference in backscattering of the laser beams, which is the variation in the amount of light reflected back toward the source. The experiment was done in triplicate to confirm the reproducibility of the results.

### 2.7 Bacterial Cell Surface Hydrophobicity—BATH Test

The bacterial adhesion to hydrocarbons (BATH) test was performed at ambient conditions (25 ± 1°C) to investigate the bacterial cell surface hydrophobicity according to the method described by Pérez et al. [18]. Three milliliter of fresh bacterial culture was diluted to an optical density measured at a wavelength of 600 nm OD_600_ = 0.6 (OD_1_), then mixed with 3 mL mineral oil (8042‐47‐5, Sigma Aldrich, St. Louis, MO, USA). The mixture was vigorously vortexed for 1 min. The biphasic system was then allowed to settle for 15 min, leading to the separation of water and oil phases. The test was performed in triplicate. The water phase was taken to measure the OD_600_ (OD_2_). The hydrophobicity of the bacterial cell surface was calculated using the following Equation (2):

(2) $$\begin{array}{ccc} & & \mathrm{BATH}\;\mathrm{hydrophobicity}\;\mathrm{of}\;\mathrm{bacterial}\;\mathrm{cell}\;\mathrm{surface}\;\left(\%\right)=\\ & & \left(1-\frac{OD_{1}-OD_{2}}{OD_{1}}\right)\times 100 \end{array}$$

### 2.8 Interfacial Tension Between Bacterial Culture and Mineral Oil

The interfacial tension between bacterial culture and mineral oil was investigated at ambient conditions (25°C) using a goniometer (DSA30, Krüss, Germany) equipped with image analysis software (Advance, Drop Shape, version 1.9, Krüss, Germany) following the pendant drop method as previously described by Rühs et al. [19]. Fresh bacterial suspension in PBS (OD_600_ = 0.6) was loaded in a syringe with a needle of 1.53 µm internal diameter. The drop was generated into mineral oil, creating a bacterial culture/mineral oil interface. The presence of bacterial cells and extracellular compounds in the drop can act as surface‐active agents, reducing the interfacial tension. The interfacial tension was determined based on the analysis of the drop shape change due to the pressure difference across a curved liquid interface according to the Young–Laplace equation:

(3) $$\Delta p=\sigma \left(\frac{1}{R_{1}}+\frac{1}{R_{2}}\right)$$

where Δp is the Laplace pressure, the pressure difference across the fluid interface (the exterior pressure minus the interior pressure in the drop), σ is the surface tension, *R*_1_ and *R*_2_ are the principal radii of curvature.

### 2.9 Surface Properties of Bacteria and Intestinal Mucus

#### 2.9.1 Preparation of Porcine Intestinal Mucus

Due to the anatomical similarities between the digestive systems of pigs and humans, the intestinal mucus of pigs was used in this study instead of the human one [20]. The preparation of porcine intestinal mucus was done following the same procedure described previously [17]. Pig digestive systems were collected at the local abattoir where meat is produced for commercial purposes, no ethical permit was required for the current study. Large and small intestine mucus from pigs were collected in separate containers and subsequently sieved through a mesh with a hole size of 0.4 mm to exclude larger particles. After being diluted twice in 10 mM HEPES (4‐(2‐hydroxyethyl)‐1‐piperazineethanesulfonic acid), the collected viscous fluid was separated from other contaminants and water was removed from the supernatant by centrifugation.

#### 2.9.2 Preparation of Bacterial and Mucus Thin Layers

One milliliter of fresh bacterial or mucus suspension in PBS solution (OD_600_ = 1.0) was deposited on a Millipore filter membrane with a pore size of 0.22 µm, 47 mm diameter (GSWP04700–Merck KGaA, Darmstadt, Germany) to create a thin layer of bacteria. The membrane was then stored at controlled conditions of 30% relative humidity and 25°C for 1 h before measurement.

#### 2.9.3 Contact Angle Measurement With Different Liquids

The contact angle of water, ethylene glycol, diiodomethane, and cyclopentanol on a thin layer of bacteria or mucus was determined at ambient conditions (25°C) according to the sessile drop method using a goniometer (DSA30, Krüss, Germany) equipped with image analysis software (Advance, Drop Shape, version 1.9, Krüss, Germany). Detailed physicochemical parameters of the four liquids used can be found in Appendix 2. A liquid drop of around 3 µL was deposited on the surface of the film. The initial contact angle after the drop was deposited on the surface was considered. At least eight replicates were performed for each liquid.

#### 2.9.4 Determination of Surface Tension of the Bacterial/Mucus Thin Layer

The polar and dispersive components of the surface tension of bacteria and mucus layers were determined according to the *Owens and Wendt* methods, using Equation (4) as follows:

(4) $$\frac{\sigma_{L}\left(\cos\Theta +1\right)}{2\sqrt{\sigma_{L}^{D}}}=\frac{\sqrt{\sigma_{S}^{P}}+\sqrt{\sigma_{L}^{P}}}{\sqrt{\sigma_{L}^{D}}}+\sqrt{\sigma_{S}^{D}}$$

A linear regression based on the properties of the liquids and the measured contact angles allows for the calculation of the dispersive ($\sigma_{S}^{D})$ and polar components ($\sigma_{S}^{P})\;$ of the solid surface which is the bacterial or mucus layer in this study. The data treatment proposed by Chanut et al. [21] has been used in order to take into account the variability induced both by experimental analysis and modeling.

#### 2.9.5 Work of Adhesion

The work of adhesion ($W_{a}$) between a layer of bacteria and a layer of mucus was calculated according to the Dupré equation as follows:

(5) $$W_{a}=2\sqrt{\sigma_{1}^{D}\sigma_{2}^{D}}+2\sqrt{\sigma_{1}^{P}\sigma_{2}^{P}}$$

where $\sigma_{1}^{D}$, $\sigma_{2}^{D}$, $\sigma_{1}^{P}$, and $\sigma_{2}^{P}$ refer to the dispersive and polar contributions of the surface tension of bacterial and mucus layers.

### 2.10 Statistical Analysis

Data are presented as mean ± standard deviation (*n* ≥ 3 biological repetitions). Statistical analysis used one‐way ANOVA with Tukey's post hoc test (*p* < 0.05). Data were processed using MATLAB 2019b.

## 3 Results and Discussion

### 3.1 Morphological Expressions of Bacteria Related to Their Adhesion Potential to the Intestinal Tract

Within the strong hydrodynamic flow of the bulk liquid inside the gastrointestinal environment, probiotic cells can move actively by swimming or passively by the traction forces of the gut, Brownian motion, and gravitational force [4]. The closer probiotics can approach the intestinal substratum, the higher the chance for adhesion to be initiated. Moreover, the interaction forces between probiotics themselves and intestinal surrounding components are significantly affected by their cell morphological properties. Accordingly, these parameters can be interesting indicators of their adhesion potential in the gut.

**FIGURE 2:**
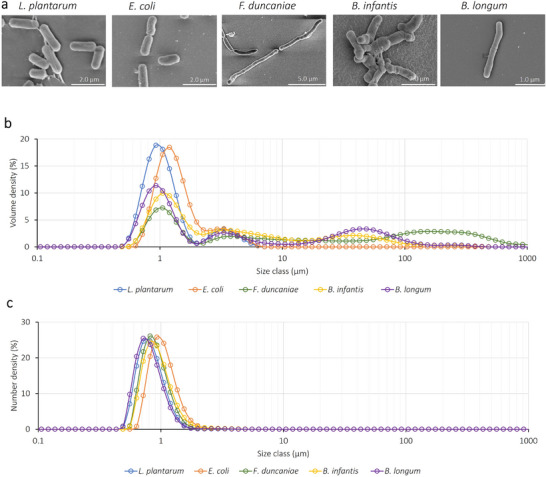
Morphological properties of the five selected bacterial strains. (a) Scanning electron microscopic images; (b) size distribution, as measured by laser light scattering, and reported in volume density; and (c) size distribution displayed in number density. The laser scattering analysis was conducted with bacterial suspensions in PBS medium, at 25 ± 1°C. The corresponding distribution represents the mean value obtained from three repetitions of three independent times.

To assess the impact of physicochemical properties on the bacterial adhesion potential to the intestinal surface, the five bacterial strains selected in the present study are all mucus‐associated bacteria [22, 23, 24, 25, 26]. The SEM images first show that these five strains possess distinguished morphological attributes (Figure 2a). *E. coli* K‐12 TGI and *Lpb. plantarum* 103151T cells are rather short and tend to separate from each other, while the remaining three strains tend to aggregate to form long chains or clusters. To further investigate the size distribution of these bacterial strains, bacterial suspensions were subjected to laser light scattering. Although such a method considers all particles as round shapes, it provides relevant information about statistical size distribution. The corresponding results are reported in volume density (Figure 2b) and number density (Figure 2c).

Figure 2b revealed that *Lpb. plantarum* 103151T and *E. coli* K‐12 TGI presented mainly as single cells with a main size distribution of around 1 µm and with a small fraction of 2–3 µm. In contrast, *B. longum* BB536, *B. infantis* S12, and *F. duncaniae* A2‐165 all showed fractions of long chains in the range of 40–50 µm, and even 200–300 µm for *F. duncaniae* A2‐165. Figure 2c reports the number density of corresponding bacterial size distribution. Such representation reveals that, for all tested strains, the number of single cells with a length of about 1 µm present in the suspension far surpasses the number of long chains previously identified using a representation in volume. Yet, in *F. duncaniae* A2‐165, *B. longum* BB536, and *B. infantis* S12, while the ratio of chains to single cells is negligible in number, these long chains occupy a substantial portion of the total volume of the cell population in the suspension. However, according to SEM investigation (Figure 2a), *B. longum* BB536 and *B. infantis* S12 cells did not form long chains of 40–50 µm, as revealed by laser light scattering. This could be attributed to the presence of exopolysaccharide (EPS) chains produced by Bifidobacteria, which is a well‐known phenomenon in this bacterial genus [27]. The formation of long chains when cells aggregate, as well as the presence of exopolysaccharides, have a positive effect on the adhesion of bacteria in the intestinal tract [28, 29, 30, 31]. Therefore, the knowledge of the size distribution of these bacteria represents a first step in the investigation of their further adhesion potential in the gut.

### 3.2 Rheological Properties of Bacteria Related to Their Adhesion Potential to the Gut

Rheological properties of probiotic bacteria are the manifestations of their morphological characteristics which reveal their adhesion potential to the gut. Sedimentation is the primary mechanism of bacterial mass transport [32, 33]. Therefore, this is a crucial step that can bring bacteria closer to the substratum [34, 35], which can allow them to initiate further interactions with the intestinal surface.

**FIGURE 3:**
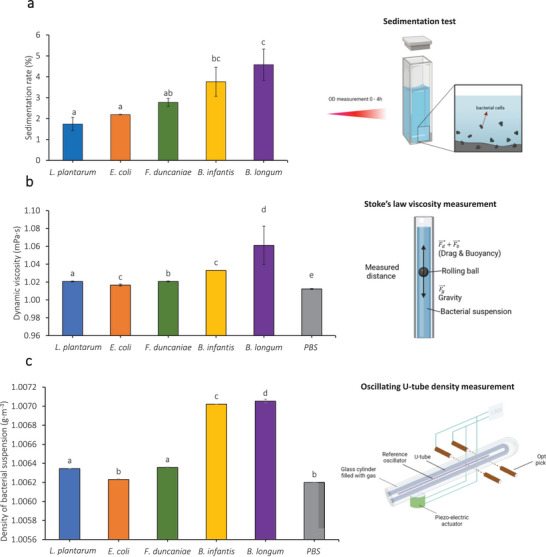
(a) Sedimentation rate of bacteria per hour; (b) dynamic viscosity, as measured using a rolling ball in the capillary method; and (c) density of bacterial suspensions based on oscillating U‐tube density method, for the strains *Lpb. plantarum* 103151T, *E. coli* K‐12 TGI, *F. duncaniae* A2‐165, *B. longum* subsp. *infantis* S12, and *B. longum* BB536. The viscosity and density of PBS were used as a control (medium without bacteria). The Latin letters distinguish the difference between the five strains for their sedimentation rate per hour using one‐way ANOVA with Tukey's test (*p* < 0.05). The corresponding data represent the mean value obtained from three repetitions of three independent times.

The sedimentation rate per hour of the five bacterial strains of interest, as determined within 4 h, ranges from 1.74% to 4.58% (Figure 3a). On the one hand, *E. coli* K‐12 TGI and *Lpb. plantarum* 103151T exhibited the slowest sedimentation, only 1.74% and 2.19%, respectively. On the other hand, *B. longum* BB536 cells settle with the highest rate among the five strains, followed by *B. infantis* S12 and *F. duncaniae* A2‐165, respectively. The previous SEM observations (Figure 2a) have pointed out that the single cells of *E. coli* K‐12 TGI and *Lpb. plantarum* 103151T do not aggregate into chains or clusters but separate from one another, as already reported in a previous study [17]. The cell separation results in a better dispersion of bacteria in the suspension, leading to the low sedimentation rate of these two strains in this test. In contrast, the high sedimentation rate of *F. duncaniae* A2‐165, *B. longum* BB536, and *B. infantis* S12 is also interestingly correlated to previous results where these bacteria exhibited an aggregation behavior [17]. The formation of chains and clusters in these three strains was also proven to facilitate the adhesion in the intestinal surface [17]. Accordingly, the higher sedimentation rate can be referred to as the higher adhesion potential of bacteria in the gut.

Moreover, Figure 3b displays the dynamic viscosity, and Figure 3c shows the density of bacterial cell suspensions. Interestingly, these two parameters correlate well with the sedimentation rate of bacteria, as reported in Figure 3a. *B. infantis* S12 and *B. longum* BB536 exhibited higher viscosity and higher density compared to the other strains. This might be due to the EPS‐forming ability of these two strains, as suspected from the size distribution analysis (Figure 2b). The higher density of *B. infantis* S12 and *B. longum* BB536 also explains their higher sedimentation rate compared to the other strains. Therefore, rheological analyses including sedimentation rate, viscosity, and density of bacterial suspension have the potential to reveal the adhesion potential of bacteria at the initial stage when probiotics enter the intestinal tract.

**FIGURE 4:**
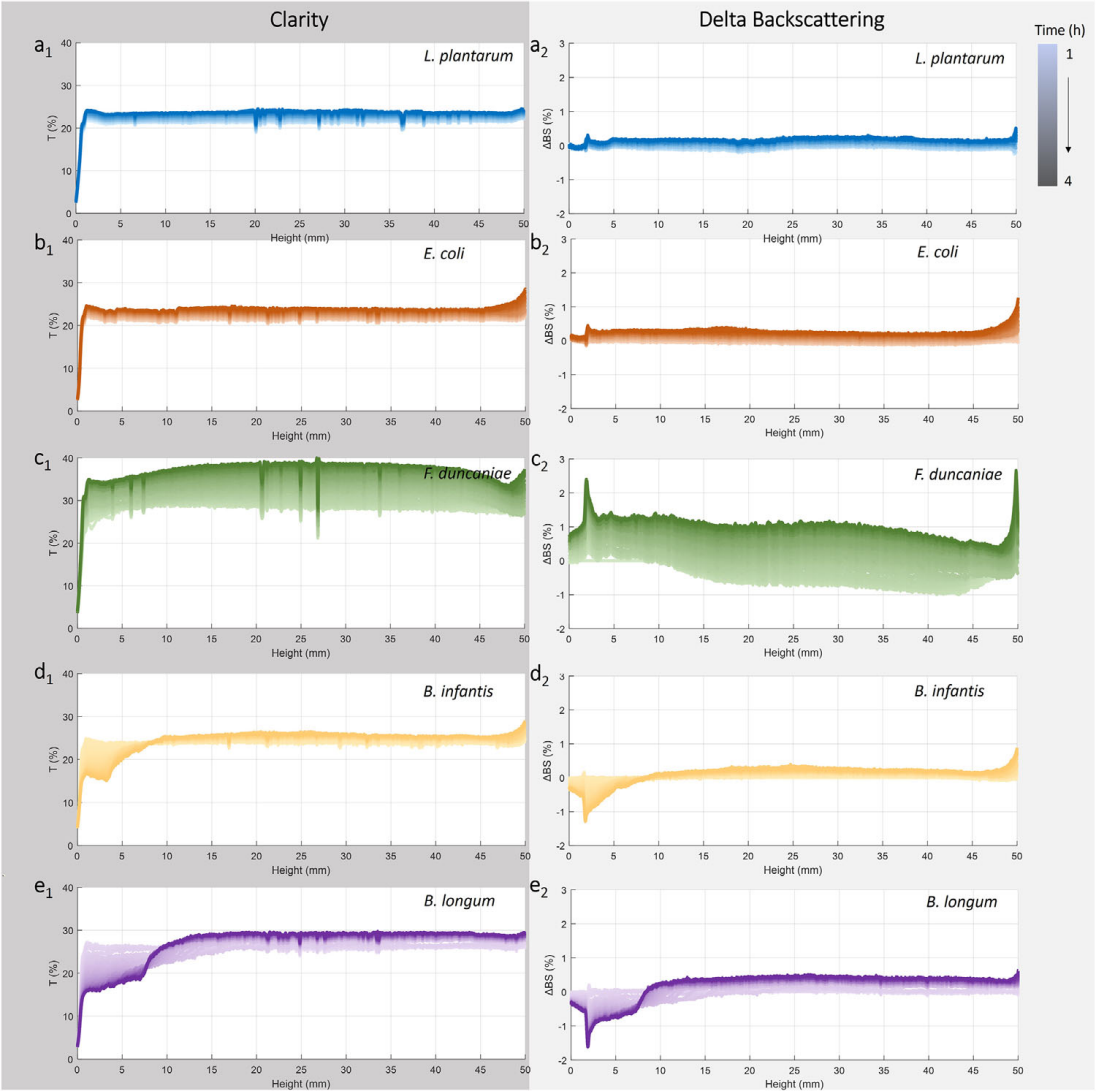
Physical destabilization of bacterial suspensions. Evolution of clarity (%*T*) and delta backscattering (ΔBS), during 4 h, at 25°C (the evolution is expressed by the change from light to dark color). (a_1_, a_2_) *Lpb. plantarum* 103151T; (b_1_, b_2_) *E. coli* K‐12 TGI; (c_1_, c_2_) *F. duncaniae* A2‐165; (d_1_, d_2_) *B. longum* subsp. *infantis* S12; and (e_1_, e_2_) *B. longum* BB536. The corresponding data represent the value obtained from one scan, which was confirmed to have similar kinetics to two other independent repetitions.

The physical destabilization of the bacterial suspension allows for understanding the changes occurring in the spatial distribution of bacterial particles over time. These parameters are correlated to the aggregating behavior and sedimentation rate, thus, affecting the adhesion. Overall, the suspensions of all five strains demonstrated an increase of the ΔBS at the top of the sample due to an augmented concentration of the disperse phase, like a cream layer (Figure 4). Besides, the occurrence of flocculation is shown by the evolution of the backscattering throughout the entire sample height due to the global increase in particle size. However, the phenomenon of flocculation exhibited greater intensity in the suspensions of *F. duncaniae* A2‐165 and *B. longum* BB536, with ΔBS increasing by 1.5% and 0.5%, respectively, compared to approximately 0.2% observed in the other three strains. This indicates a strong aggregation of cells for the two strains *F. duncaniae* A2‐165 and *B. longum* BB536, resulting in a progressive enlargement of particle size over time. It is correlated with the previously reported higher sedimentation rate for these two strains (Figure 3a). In addition, other destabilizing phenomena were also observed at the bottom of the suspensions. Specifically, *Lpb. plantarum* 103151T, *E. coli* K‐12 TGI, and *F. duncaniae* A2‐165 all demonstrated an elevation in backscattering at the bottom, which indicates a sedimentation phenomenon. In contrast, *Bifidobacterium* suspensions exhibit a clarification phenomenon at the bottom, attributed to pronounced creaming‐like behavior. This occurrence might be explained by the production of exopolysaccharides by these two strains, which also induced a higher sedimentation rate, as previously reported. The physical destabilization is related and is indeed the expression of morphological properties of bacterial cells in suspension, which play crucial roles in the interaction and adhesion of bacteria in the gut at the transport stage. For these reasons, the investigation of the physical destabilization of bacterial suspensions also represents a useful approach to studying the bacterial adhesion potential in the gut.

### 3.3 Hydrophobicity, Interfacial Behavior, and Adhesion Potential of Bacteria in the Gut

Upon reaching closer to the intestinal substratum, the bacterial reversible adhesion is governed by various factors such as Lifshitz–van der Waals forces, electrostatic forces, and the hydrophobic properties of the bacterial cell as well as the intestinal mucus surfaces [6, 34]. However, as mentioned previously, the impact of the hydrophobic interactions is much more significant than the other forces. Therefore, in the present work, the hydrophobicity and surface properties of bacterial cells surface and porcine intestinal mucus were taken into investigation. It is commonly admitted that hydrophilic cells strongly adhere to hydrophilic surfaces, whereas hydrophobic cells adhere more to hydrophobic surfaces [36, 37]. Considering the highly hydrophobic nature of the intestinal mucus according to previous studies, the hydrophobicity of bacterial cell surface has been considered one of the crucial parameters that could facilitate the initial bacterial interaction and then adhesion to solid surfaces including biomaterials and animal cells [30, 38]. However, some previous studies have shown no correlation between bacterial cell surface hydrophobicity and their adhesion to the intestinal mucus layer [39, 40]. This led to concerns regarding the hydrophobic hypothesis of the intestinal mucus previously proposed. Accordingly, in this study, a comprehensive experimental series was carried out including BATH hydrophobicity, surface tension of bacterial and mucus thin layer, and interfacial tension of bacterial solution and mineral oil, where both bacteria and porcine intestinal mucus are evaluated.

**FIGURE 5:**
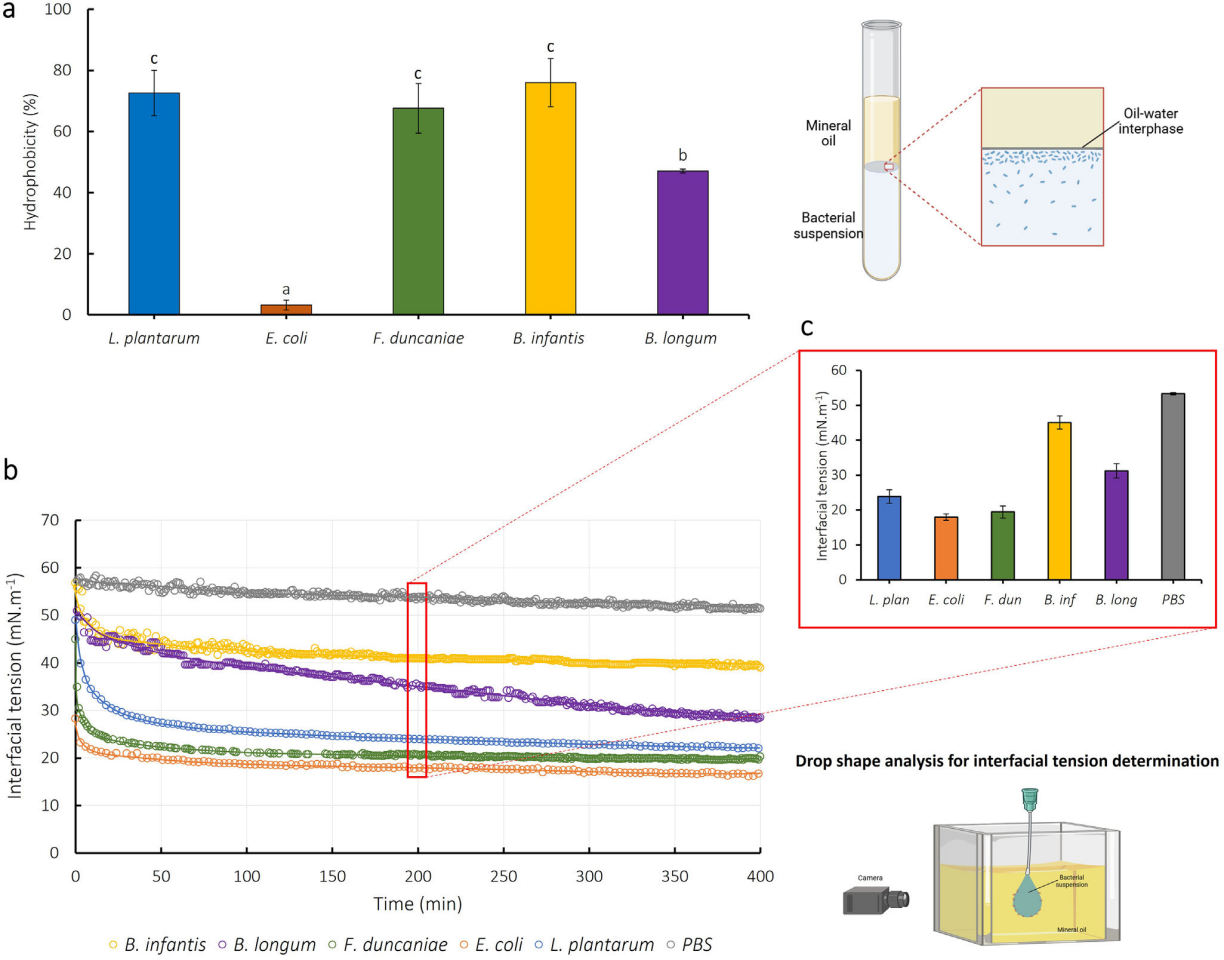
(a) Hydrophobicity of the five bacterial strains: *Lpb. plantarum* 103151T, *E. coli* K‐12 TGI, *F. duncaniae* A2‐165, *B. infantis* S12, and *B. longum* BB536 according to the bacterial adhesion to hydrocarbons test (also referred to as BATH test). The detailed data of BATH hydrophobicity of five strains can be found in Appendix 1. The Latin letters a, b, and c distinguish the difference between the five strains for their cell hydrophobicity, based on one‐way ANOVA using Tukey's test (*p* < 0.05). (b) Interfacial tension between mineral oil and bacterial culture of five bacterial strains: *Lpb. plantarum* 103151T, *E. coli* K‐12 TGI, *F. duncaniae* A2‐165, *B. longum* BB536, *B. infantis* S12 and PBS used as the blank medium measured according to the pendant drop method; and (c) interfacial tension of bacterial culture and mineral oil at 200 min. The corresponding data represent the mean value obtained from three repetitions of three independent times.

To evaluate the hydrophobicity of bacterial cell surface, the bacterial adhesion to hydrocarbons (BATH) and interfacial tension (IFT) measurement based on the pendant drop method were performed on the five bacterial strains of interest using mineral oil as the hydrophobic phase. According to Figure 5a, *Lpb. plantarum* 103151T, *F. duncaniae* A2‐165, and *B. longum* BB536 exhibited similar high hydrophobicity, which was in the range of 66.7%–76.0%. In contrast, *E. coli* K‐12 TGI showed very low hydrophobicity compared to the other strains, which was around 3.2%. *B. longum* BB536 cell hydrophobicity ranged in the middle, with a value of 47.1%.

The hydrophobicity of bacterial cell surface depends on various factors including the composition of the cell wall; the presence of fimbriae, pili and/or appendages, cell membrane hydrophobic proteins, lipids or fatty acids; as well as the presence of exopolysaccharides and cell surface charge [41]. Further molecular analysis would be required to go deeper in the relationship between the surface composition of bacterial cells and their hydrophobicity.

Compared to the results from the previous study [17], the BATH hydrophobicity of five selected strains is not correlated with the agglomeration and the adhesion ability of bacterial cells to the intestinal mucus. *E. coli* K‐12 TGI displayed only 3.2% of hydrophobicity according to the BATH test, which is the lowest among the five tested strains, but showed the highest agglomeration capacity to porcine intestinal mucus. *F. duncaniae* A2‐165 possesses a high BATH hydrophobicity but low agglomeration to porcine intestinal mucus. Therefore, the BATH hydrophobicity does not provide relevant enough information related to the adhesion potential of bacteria in the intestinal tract. Accordingly, the interfacial tension (IFT) between bacterial suspension and mineral oil was also investigated.

Figure 5b exhibits the IFT decrease over time between mineral oil and bacterial cultures of the five strains of interest, as measured by the pendant drop method. Overall, all tested bacteria displayed a decreasing trend on the IFT between the two phases compared to the controlled sample of PBS, which corresponds to the blank medium without the presence of bacteria. The bacteria cells act as biosurfactants at the water/oil interface, thus reducing the IFT between the aqueous and mineral oil phases [42]. The IFT of *Lpb. plantarum* 103151T and *F. duncaniae* A2‐165 dropped approximately to 25 mN·m^−1^ within the first 50 min of the test, which was the strongest decrease observed among the five strains. For *E. coli* K‐12 TGI, *B. infantis* S12, and *B. longum* BB536, the reduction was only around 10–15 mN·m^−1^ at that stage. After the first 100 min of the test, the IFT values of all strains reached an equilibrium, except for *B. longum* BB536, whose IFT continued to decrease significantly at a rate of 0.04 mN·m^−1^·min^−1^. This could be due to the gravity and sedimentation effect of *B. longum* BB536 cells compared to the other strains. After 400 min, the IFT of *E. coli* K‐12 TGI, *Lpb. plantarum* 103151T, and *F. duncaniae* A2‐165 reached relatively low value, around 20–25 mN·m^−1^, while they were 30 and 40 mN·m^−1^ for *B. longum* BB536 and *B. infantis* S12, respectively. Figure 5c exhibits the IFT values of bacterial suspensions or PBS with mineral oil at 200 min of the test. Higher IFT was observed in the *B. infantis* S12 and *B. longum* BB536 suspensions, which follows the same tendency as for rheological properties. However, no correlation was found between IFT and BATH hydrophobicity.

The different IFT observed for the different bacterial strains are strongly affected by various factors including the morphology and the surface components of the bacterial cells. *E. coli* cells are covered by pili, which are believed to play a significant role in hydrophobic interactions [43]. This is correlated to the low IFT of *E. coli* K‐12 TGI measured in the present study. However, the IFT may also be affected by many other factors. The presence of cell wall peptidoglycans, teichoic acids, lipoteichoic acids, and lipopolysaccharides on the bacterial surface could potentially play the role of surfactants and thus affect the IFT [44]. Accordingly, this parameter does not reflect only the impact of bacterial surface hydrophobicity.

The governing principle to evaluate the bacterial adhesion to the intestinal tract has been based on the cell surface hydrophobicity, relying on the hypothesis of the hydrophobic nature of the intestinal mucus layer. However, mucus is a highly complex system whose hydrophobicity is affected by several components, including glycoproteins, polysaccharides, lipids, salts, and proteins [45]. It is, hence, necessary to study the surface properties of both bacteria and intestinal mucus for a better understanding of their affinity to each other. Thus, the surface tension of bacteria and mucus layers based on contact angle measurement and thereby the calculation of their work of adhesion were proposed in the present work as an alternative approach to study bacterial adhesion to the intestinal tract.

**FIGURE 6:**
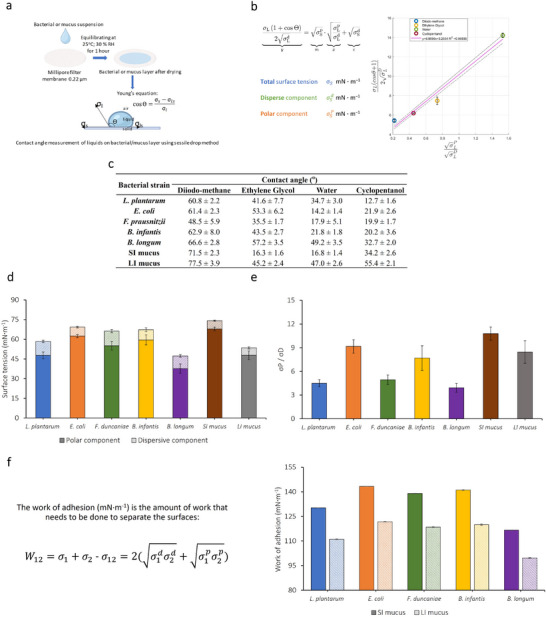
Surface tension and work of adhesion of bacteria and porcine intestinal mucus. (a) Schematic illustration of the contact angle measurement on a thin layer of bacteria or mucus; (b) principle for the calculation of the surface tension including dispersive and polar components, based on the contact angle values obtained from the sessile drop method using the *Owens and Wendt* equation (the Owens and Wendt's plot for the determination of the dispersive and polar components of the surface tension of bacteria and porcine intestinal mucus can be found in Appendix 3); (c) contact angles measured with four liquids: diiodo‐methane, ethylene glycol, water and cyclopentanol on a thin layer of one of the five bacteria of interest (*Lpb. plantarum* 103151T, *E. coli* K‐12 TGI, *F. duncaniae* A2‐165, *B. infantis* S12, and *B. longum* BB536); or on a thin layer of mucus from small (SI mucus) or large (LI mucus) intestine of the pig, the thin layers of bacteria and mucus were prepared from three different biological replicates, the contact angle values represent the mean from at least 10 measurements; (d) surface tension of bacterial and intestinal mucus layers with corresponding polar and dispersive components (mN·m^−1^) (detailed values of surface tension of five bacteria of interest and mucus can be found in Appendix 4); (e) ratio of polar (*σ* ^P^) over dispersive (*σ* ^D^) component of the surface tension of bacterial or mucus layers; and (f) work of adhesion between bacterial and SI or LI mucus layers (mN·m^−1^), as calculated from the Dupré equation using polar and dispersive components of the surface tension (detailed values of the work of adhesion of five bacteria of interest and mucus can be found in Appendix 5).

Overall, the total surface tension of all the bacterial strains tested and intestinal mucus types falls in a rather high range from 47 to 74 mN·m^−1^ (Figure 6d). Moreover, all the samples, including bacteria and mucus, exhibited a higher polar component of the surface tension than the dispersive one. The *σ* ^P^/*σ* ^D^ ratio is in the range of 4–11, as reported in Figure 6e. Among the five bacterial strains, *E. coli* K‐12 TGI cells presented the highest *σ* ^P^/*σ* ^D^ ratio, which is correlated with the lowest BATH hydrophobicity (Figure 5a). This result additionally proves that even though bacteria and mucus all present both hydrophobic and hydrophilic domains, they are mostly hydrophilic.

The work of adhesion is defined as the reversible thermodynamic work, that is, needed to separate the interface from the equilibrium state of two phases to a separation distance of infinity [46]. This calculation was applied using the previously measured surface tension components of bacterial and mucus surfaces. This parameter could be used as a novel indicator for the adhesion potential of bacteria to the gut. The work of adhesion of bacteria to small intestinal mucus was overall higher than to large intestinal mucus (Figure 6f). This corresponds to the fact that the *σ* ^P^/*σ* ^D^ ratio of small intestinal mucus is higher than that of large intestinal mucus. That means the more hydrophilic the bacteria is, the higher the work of adhesion with mucus. By comprehensively evaluating the surface properties of both bacterial cells and intestinal mucus, the contact angle measurement and thus work of adhesion calculation are proven to be a promising novel approach to investigate bacterial adhesion to mucus.

## 4 General Discussion

The bacterial adhesion phenomenon in the gut is governed by various factors including the bacterial cell properties and the intestinal environment [4, 47, 48]. This study combines different physicochemical approaches to elucidate bacterial adhesion potential from the initial stages of transport to the reversible adhesion in the gut. To comprehensively assess the entire adhesion process, from transport to irreversible adhesion in the mucus layer, we have also integrated results from our previous ex vivo studies on bacteria–mucus irreversible adhesion, specifically bacteria‐mucus agglomeration and viable bacteria–mucus adhesion indexes [17].

**FIGURE 7:**
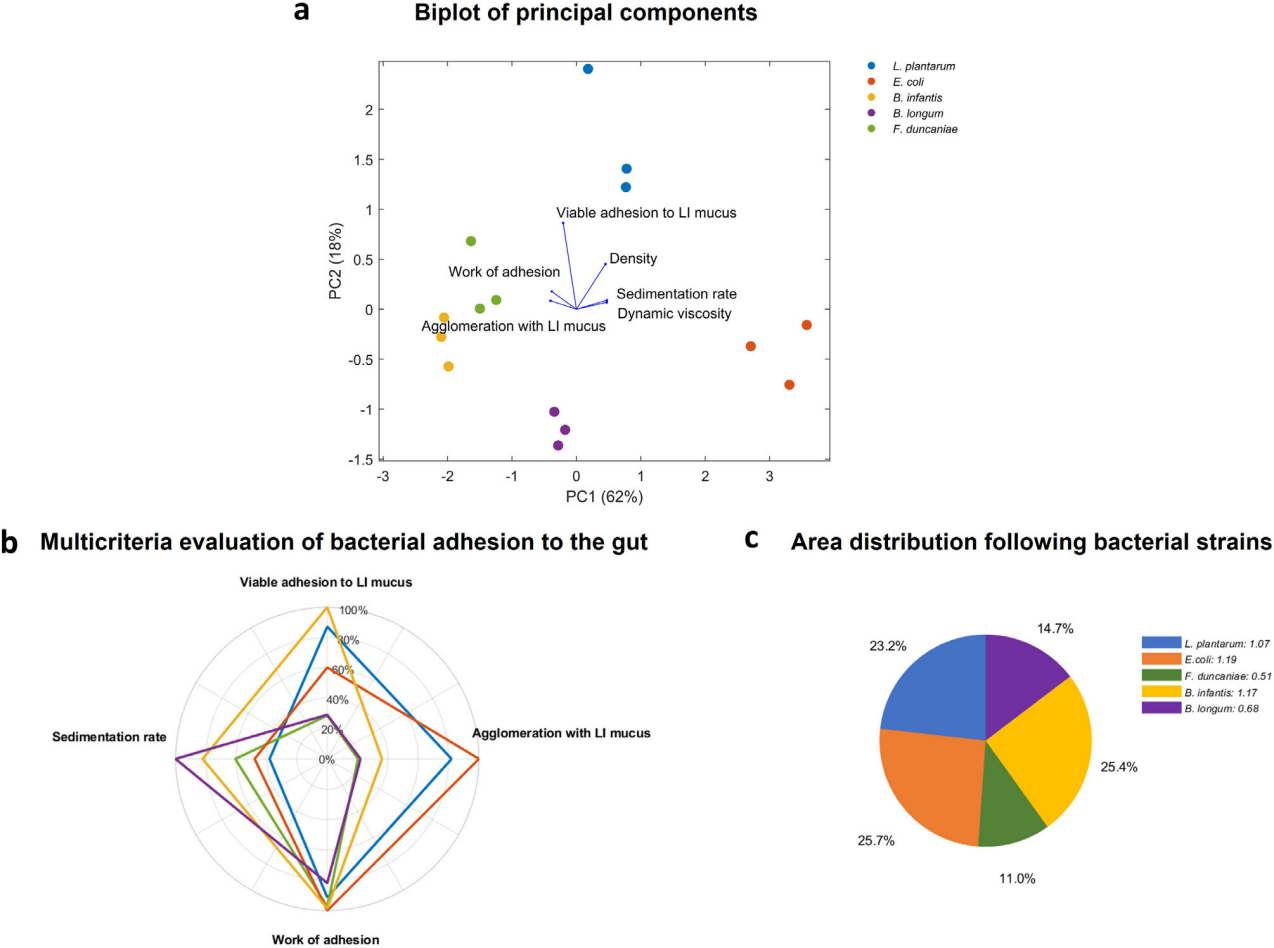
(a) Biplot of principal component analysis. (b) Multicriteria evaluation of bacterial adhesion potential to the intestinal tract. The four most critical criteria selected, including sedimentation rate, work of adhesion of bacteria and mucus, bacteria–mucus agglomeration (results obtained from our previous study [17]), and viable bacteria–mucus adhesion (results obtained from our previous study [17]). The data were normalized to percentages based on the highest values (referred to as 100%). (c) Area distribution according to bacterial strains. The area was calculated based on the multicriteria evaluation from figure (b). LI mucus indicates large intestinal mucus.

Figure 7a presents a biplot of principal component analysis (PCA), integrating bacterial cell density, viscosity, sedimentation rate, work of adhesion to mucus, viable adhesion to large intestinal (LI) mucus, and agglomeration to LI mucus. The results indicate a strong correlation between the bacteria–mucus agglomeration index and the work of adhesion derived from the current physicochemical approach, validating the proposed novel method. Morphological parameters such as sedimentation, density, and viscosity also exhibited good correlation. These parameters can independently represent adhesion potential during the transport stage. However, the viable adhesion of bacteria to LI mucus did not follow the same trend as the other parameters.

For a comprehensive evaluation, we have selected four critical parameters to score bacterial adhesion potential to intestinal mucus: sedimentation rate (representing adhesion potential during transport), work of adhesion (representing reversible adhesion potential), and agglomeration and viable adhesion indexes (representing irreversible adhesion potential). Figure 7b illustrates a radar chart based on the normalization of values, with the highest values of each parameter set at 100%. The area related to each bacterial strain in Figure 7b was calculated and expressed as a percentage, leading to the corresponding pie chart as shown in Figure 7c. The results reveal that *E. coli* K‐12 TGI and *B. infantis* S12 demonstrate the highest overall adhesion potential in the intestinal tract, with areas calculated at 1.19 and 1.17, respectively. This is followed by *Lpb. plantarum* 103151T with an area of 1.07. *B. longum* BB536 and *F. duncaniae* A2‐165 exhibited the lowest areas at 0.68 and 0.51, respectively. However, it is important to note that this evaluation method is inherently relative. Depending on the selected method used to determine each single parameter, the assessment of overall adhesion potential may vary. Further studies may develop additional evaluation methods. For instance, the scoring for viable adhesion can be improved by considering the average number of single cells within chains or clusters, particularly in the case of *F. duncaniae*, *B. longum*, and *B. infantis*. This would provide a more accurate quantification compared to the CFU method. Nonetheless, such scoring method provides a comprehensive evaluation of the total adhesion potential of probiotic bacteria to the intestinal tract.

## 5 Conclusions

This work provides a comprehensive understanding of the bacterial adhesion phenomenon in the gut by introducing novel physicochemical approaches to evaluate bacterial adhesion to the intestinal tract. Morphological and rheological analyses of bacterial cells serve as indicators of their adhesion potential at the initial stage of transport within the bulk liquid flow of the intestinal cavity. Surface analysis of both bacterial cells and intestinal mucus, conducted via contact angle measurements, allows for the calculation of the work of adhesion between these components. This parameter has also been shown to be highly correlated with the bacteria‐mucus agglomeration index investigated in the previous work.

This work provides evidence that bacterial hydrophobicity, as measured by the Bacterial Adhesion to Hydrocarbons (BATH) and interfacial tension tests, is not relevant to the adhesion potential of bacteria to mucus. This, additionally, led to a re‐examination of the hydrophobic hypothesis of intestinal mucus, a fundamental concept underlying previous hydrophobicity tests. Contrary to expectations, the results show that intestinal mucus is not hydrophobic but rather hydrophilic, tending to adhere better to hydrophilic bacteria.

A multicriteria evaluation of full‐scale bacterial adhesion potential to the intestinal tract was performed by integrating results from the relevant physicochemical analysis in this study with previous ex vivo approaches including bacteria–mucus agglomeration and viable bacteria–mucus adhesion indexes. The findings indicate the adhesion potential of five tested bacterial strains ranks in the following order: *E. coli* K‐12 TGI, *B. infantis* S12, *Lpb. plantarum* 103151T, *B. longum* BB536, and *F. duncaniae* A2‐165.

Coupling such physicochemical approaches therefore shows great promise for investigating the bacterial adhesion phenomenon in the intestinal tract, providing complementary insight to the traditional biological tests. Understanding comprehensively the adhesion potential of probiotics in the gut allows for further development of strategies to improve the adhesion of bacteria to the host. These methods could also aid in selecting appropriate probiotic bacterial strains to ensure the long‐term effects of probiotic supplementation.

## Conflicts of Interest

The authors declare no conflicts of interest.

## Supporting information



Supporting Information

## Data Availability

The data that support the findings of this study are available from the corresponding author upon reasonable request.
