# Supplementary material for: Unlocking Probiotic Potential: Physicochemical Approaches to Evaluate Probiotic Bacterial Adhesion Potential to the Intestinal Tract
Source: Mol Nutr Food Res. 2025 Jan 23;69(17):e202400705. doi: 10.1002/mnfr.202400705 (PMC12410510; doi:10.1002/mnfr.202400705)
Supplement: Supplementary file 1 — Supporting Information [file MNFR-69-e202400705-s001.docx]

**Supplementary materials**

**Appendix 1.** BATH hydrophobicity of cell surface of 5 bacterial strains of interest: *L. plantarum*, *E. coli*, *F. duncaniae*, *B. infantis* and *B. longum*.

|  | **Hydrophobicity** |
| --- | --- |
| ***L. plantarum*** | 72.6±7.5^a^ |
| ***E. coli*** | 3.2±1.6^b^ |
| ***F. duncaniae*** | 67.6±8.1^a^ |
| ***B. infantis*** | 76.0±7.9^a^ |
| ***B. longum*** | 47.1±0.7^c^ |

**Appendix 2.** Physicochemical properties of the liquids used for surface tension measurement (at 25 °C).

| **Liquids** | **Density (g.cm^-3^)** | **Total surface tension (mN.m^-1^)** | **Polar part (mN.m^-1^)** | **Dispersive part (mN.m^-1^)** | **Reference** |
| --- | --- | --- | --- | --- | --- |
| **Diiodo-methane** | 3.3 | 50.8 | 2.3 | 48.5 | [1], [2] |
| **Ethylene Glycol** | 1.109 | 47.7 | 16.8 | 30.9 | [1], [3] |
| **Water** | 0.998 | 72.8 | 51 | 21.8 | [3], [4] |
| **Cyclopentanol** | 0.948 | 32.7 | 5.5 | 27.2 | [5], [6] |

**Appendix 3.** *Owens and Wendt*'s plot for the determination of the dispersive and polar components of the surface tension of bacteria and porcine intestinal mucus included the variability related to experimental data and modeling. Each data point was calculated using the contact angles of 4 liquids (Diiodo-methane, ethylene glycol, water and cyclopentanol) measured using the sessile drop method. The plots were created using a specific MATLAB program developed by Chanut *et al.* 2022[7] where the variability induced both by experimental analysis and modeling were taken into account.

**Appendix 4.** Surface tension of 5 bacterial of interest, *L. plantarum*, *E. coli*, *F. duncaniae*, *B. infantis* and *B. longum*, and mucus collected from small and large intestine of pigs based on contact angle measurement on bacterial or mucus layers.

|  | **Surface tension (mN.m^-1^)** | | | **σ^P^ / σ^D^** |
| --- | --- | --- | --- | --- |
|  | **Polar component (σ^P^)** | **Dispersive component (σ^D^)** | **Total surface tension (σ^P^+σ^D^)** |  |
| *L. plantarum* | 47.8±2.6 | 10.6±0.9 | 58.4±2.1 | 4.5±0.5 |
| *E. coli* | 62.5±1.2 | 6.8±0.6 | 69.3±0.9 | 9.2±0.9 |
| *F. duncaniae* | 55.1±3.3 | 11.2±1.2 | 66.2±2.3 | 4.9±0.6 |
| *B. infantis* | 59.6±3.8 | 7.8±1.5 | 67.3±2.4 | 7.7±1.6 |
| *B. longum* | 37.7±3.6 | 9.6±1.0 | 47.3±2.8 | 3.9±0.6 |
| *SI mucus* | 67.9±1.4 | 6.3±0.5 | 74.1±0.9 | 10.8±0.8 |
| *LI mucus* | 47.8±3.3 | 5.7±0.9 | 53.5±2.6 | 8.5±1.4 |

**Appendix 5.** Work of adhesion of bacteria to mucus calculated using the Dupré equation with the values of polar and dispersive surface tensions of 5 bacterial strains and porcine intestinal mucus.

| **Bacterial strain** | **Work of adhesion of bacteria to mucus (mN.m^-1^)** | |
| --- | --- | --- |
|  | **Small intestine** | **Large intestine** |
| *L. plantarum* | 130.2±0.1 | 111.1±0.3 |
| *E. coli* | 143.3±0.1 | 121.7±0.2 |
| *F. duncaniae* | 139.0±0.1 | 118.5±0.3 |
| *B. longum* | 141.1±0.2 | 120.0±0.4 |
| *B. infantis* | 116.6±0.2 | 99.6±0.4 |

**Supplementary references**

[1] D. R. Lide, *CRC Handbook of Chemistry and Physics*, 80th ed. 1999.

[2] F. M. Fowkes, “Attractive forces at interfaces,” *Ind Eng Chem*, vol. 56, no. 12, pp. 40–52, Dec. 1964, doi: 10.1021/IE50660A008.

[3] G. Ström, M. Fredriksson, and P. Stenius, “Contact angles, work of adhesion, and interfacial tensions at a dissolving Hydrocarbon surface,” *J Colloid Interface Sci*, vol. 119, no. 2, pp. 352–361, Oct. 1987, doi: 10.1016/0021-9797(87)90280-3.

[4] M. Williams and M. J. O’Neil, *The Merck Index: An Encyclopedia of Chemicals, Drugs, and Biologicals, 15th Edition*, vol. 74, no. 5. John Wiley & Sons, Ltd, 2013. doi: 10.1002/DDR.21085.

[5] K. F. Gebhardt, *Grundlagen der physikalischen Chemie von Grenzflächen und Methoden zur Bestimmung grenzflächenenergetischer Größen*. 1982.

[6] D. R. Lide, *CRC Handbook of Chemistry and Physics*, 88th ed. 2007.

[7] J. Chanut *et al.*, “Surface properties of cork: Is cork a hydrophobic material?,” *J Colloid Interface Sci*, vol. 608, pp. 416–423, Feb. 2022, doi: 10.1016/J.JCIS.2021.09.140.
